# Development and diversity of Andean-derived, gene-based microsatellites for common bean (*Phaseolus vulgaris *L.)

**DOI:** 10.1186/1471-2229-9-100

**Published:** 2009-07-31

**Authors:** Matthew W Blair, Monica Muñoz Torres, Martha C Giraldo, Fabio Pedraza

**Affiliations:** 1 CIAT – International Center for Tropical Agriculture, Cali, Colombia; 2 Current address: Department of Biology, Georgetown University, Washington DC, USA; 3 Current address: Department of Plant Pathology, Kansas State University, Manhattan KS, USA; 4 Current address: Department of Plant Sciences, North Dakota State University, Fargo ND, USA

## Abstract

**Background:**

Gene-based (genic) microsatellites are a useful tool for plant genetics and simple sequence repeat loci can often be found in coding regions of the genome. While EST sequencing can be used to discover genic microsatellites, direct screening of cDNA libraries for repeat motifs can save on overall sequencing costs. The objective of this research was to screen a large cDNA library from and Andean common bean genotype for six di-nucleotide and tri-nucleotide repeat motifs through a filter hybridization approach and to develop microsatellite markers from positive clones.

**Results:**

Robotics were used for high-throughput colony picking and to create a high-density filter of 18,432 double spotted cDNA clones which was followed by hybridization with repeat motif containing probes based on GA, CA, AAT, CAG, CAA and ACG repeats. A total of 1203 positive clones were identified by their addresses and sequenced from 5' ends and if required from 3' ends to confirm repeat motif and length. Out of 886 high quality sequences, 497 had complete microsatellite loci that were not truncated by the sequencing reaction and of these tri-nucleotide repeats were more common than di-nucleotide repeats. Different motifs were found in different frequencies in the 5' and 3' ends of the cDNAs. In a microsatellite development program, primers were designed for 248 SSR loci which were tested on a panel of 18 common bean genotypes to determine their potential as genetic markers finding higher average polymorphism information content for di-nucleotide repeat markers (0.3544) than for tri-nucleotide repeat markers (0.1536).

**Conclusion:**

The present study provides a set of validated gene-based markers for common bean that are derived from G19833, an Andean landrace that is an important source of disease and abiotic stress tolerance which has been used for physical map development and as a mapping parent. Gene-based markers appear to be very efficient at separating divergent wild and cultivated accessions as well as Andean and Mesoamerican genepools and therefore will be useful for diversity analyses and for comparative and transcript mapping in common bean.

## Background

Microsatellites are molecular markers based on simple sequence repeat (SSR) loci that can be isolated from both coding and non-coding regions of the genome [1]. When SSR loci are located within or near genes their contraction and expansion can affect gene expression or lead to amino acid substitutions or knock-out mutation in open reading frames (ORFs) [2]. Gene-based microsatellites have been found at a predictable frequency in gene coding regions of many organisms and are often mined from expressed sequence tag (EST) sequences [3,4] although they can also be isolated by various direct laboratory methods from cDNA libraries. Given their source, gene-based microsatellites are often called genic microsatellites or alternatively cDNA or EST-derived simple sequence repeats.

Genic microsatellites are distinguished from genomic microsatellites by being related to the transcriptome [4]. In other words, genic microsatellites may be found within coding and/or regulatory regions of genes such as 5' and 3' un-translated region and promoters, where they may sometimes affect gene expression. In contrast, genomic microsatellites are usually developed by screening of random DNA fragments from throughout the genome, most of which is non-transcribed. Genic microsatellites are often located in introns, and un-translated regions of sequenced cDNAs but can also be located in exons and in translated regions of ORFs [2]. In the latter case, alleles of genic microsatellites can be associated with structural mutations that lead to novel proteins that are larger or smaller than those of the original alleles and which can have substituted or repeated amino acids. Gene-based microsatellites have now been developed for a number of crop species, mostly based on EST sequencing projects [5-10]. The alternative approach of hybridizing SSR motif probes to cDNA libraries is another source of genic microsatellites, but in comparison is not widely used although it has the benefit of being a more directed method of discovering microsatellites within genes.

Among the food legume crops known as pulses, common bean is the most extensive and widely consumed [11]. After originating and being domesticated in two regions of the New World, giving rise to an Andean and a Mesoamerican genepool, this crop spread to many secondary centers of diversity based on its wide adaptability as a species. Common bean has one of the smallest and simplest, non-duplicated genomes among the legumes and is related to other tropical legumes in terms of genome structure and gene orthology although it can only be crossed with a few cultivated relatives [12]. Within common bean as a species, genepool distinctions between Andean and Mesoamerican beans are notable with seed size being a major distinguishing characteristic along with molecular marker polymorphism [13]. Consumer preferences, dissimilar growing conditions and incompatibility genes maintain these as separate groupings.

A small number of genic microsatellites have been developed for common bean by searching gene database sequences as was done by Yu et al. [14,15] and Blair et al. [16] and by mining an EST collection as was done by Hanai et al. [17]; however the current number does not exceed 100 independent loci. The source genotypes for the genic microsatellites was variable in the case of the GenBank derived microsatellites while for the IAC-UNA microsatellites designed by Hanai et al. [17] a Brazilian small seeded (Mesoamerican genepool) genotype was used. To date, genic microsatellites have not been developed from a large-seeded (Andean genepool) source genotype and the polymorphism detected by gene-based microsatellites has been moderate but biologically significant in distinguishing genepools [13,17]. Compared to gene-based microsatellites there is now a large number of genomic microsatellites [17-24] that have been derived from both Andean and Mesoamerican common bean sources and many of which have been genetically mapped [16,25,26]. It is notable that the genic microsatellites were found to be useful for genetic mapping in common bean because they were significantly less clustered than genomic microsatellites [16,24].

In terms of diversity assessment, Blair et al. [13] found genic microsatellites, albeit a small sample of them (< 70), to be of approximately one third lower diversity than genomic microsatellites as measured in terms of polymorphism information content, but still found them to be very useful for separating genepools and races. According to these authors, a similar sample of Andean-derived genomic microsatellites in the BM series have been highly diverse in the Andean genepool but less so in the Mesoamerican genepool. Meanwhile, genic microsatellites have been mainly developed from Mesoamerican genotypes [17] but not from Andean genotypes. Some ascertainment bias has been found in diversity analysis of genomic microsatellites with the markers developed from one genepool being more diverse in that genepool than in the other genepool of common bean. However, this has not yet been confirmed for genic microsatellites or for Andean-derived microsatellites. In summary, gene-based microsatellites appear to be especially useful for genetic analysis of common bean and it would be ideal to have a larger set of these markers for comparative mapping and functional diversity analysis especially those from Andean sources.

The objective of this research, therefore, was to increase the number of gene-based microsatellites through a cDNA library screening approach followed by germplasm screening. For this, multiple di and tri-nucleotide repeat containing probes were used to screen for SSR containing genes from a large scale cDNA library developed from multiple source tissues of an Andean genepool genotype (G19833 from race Peru). An Andean genepool genotype was also used as they have been less well studied than Mesoamerican beans for which a range of EST collections have been generated. Newly-developed microsatellite markers were evaluated on a diversity panel to determine their utility in diversity assessment.

## Methods

### Library construction

Library development was as described in Ramírez et al. [27]. Briefly the library was made from total RNA prepared from common bean leaves and vegetative tissue of three week old plants of the Andean cultivar 'G19833', grown in a greenhouse at the International Center for Tropical Agriculture (CIAT) under 12 hour photoperiod and average night/day temperatures of 20°C/28°C. G19833 is a Peruvian landrace from the Andean genepool that produces large, elongated, yellow seed with red mottles. The cDNA library was made from poly(A) RNA that was purified using oligo (dT) cellulose and reverse transcribed with MNLV reverse transcriptase and oligo (dT) primer. The double stranded cDNA was synthesized with polymerase I ribonuclease H co-incubation and directionally cloned into the *Not*I/*Sal*I sites of the plasmid vector, pCMVSport6.0 from Invitrogen Life Technologies (Carlsbad, CA) using DNA ligase. The library, now named PV_GEa was transformed into E. coli EMDH12S cells which were plated at a density of 3000 cfu per plate onto Q-plates with Carbenicillin (300 μg/mL), x-gal and IPTG [26]. A Q-Bot robot (Genetix, Boston, MA, USA) and automatic blue/white screening was used to pick colonies and these were arrayed into plates also by the robot. All the clones were placed into 384-well plate format glycerol stocks, grown overnight and stored at -80°C. The master plates for the library were copied twice into working copies of the library. The Q-bot was then used to spot the clones, with a double replicate 4 × 4 pattern, onto a gridded Hybond N+ membrane filter containing 6 fields of 8 × 384-well plates for a total of 48 plates.

### Filter hybridization and library screening

Six simple sequence repeat motif oligonucleotide probes were used to screen the cDNA library filters. The probes included two that targeted di-nucleotides repeat motifs, namely (CA)_20 _and (GA)_20_; and four that targeted tri-nucleotide repeat motifs, namely (AAT)_14_; (CAG)_14_; (CAA)_14_; and (ACG)_14_, where the sequence within the parenthesis indicates the repeat and the number outside the parenthesis indicates the number of copies of that repeat. Probe preparation and hybridization consisted in end labeling the simple sequence repeat motif probes with T4 DNA Kinase and hybridizing the mixture to the DNA contained on the filters with standard protocols from Edwards et al. [28]. Briefly the filters were pre-hybridized in 100 mL of hybridization buffer for 4 – 6 hours at 60°C. Meanwhile, 10 pmoles of probe was end-labeled with 1 ul of T4 polynucleotide kinase (7 Unit/ul) and 5 ul of γP^32^-ATP in a total reaction volume of 20 ul that was incubated at 37°C for 80 minutes and stopped at 65°C for 20 minutes. The labeled oligo-nucleotide was added directly to the pre-hybridizing filters and incubated at 60°C for 12 hours.

Both the di-nucleotide motif probes were grouped together for filter hybridization as were all the tri-nucleotide motif probes. After the hybridization step, the filters were washed twice at 60°C with 6× SSC/0.1% SDS for 5 minutes each. Longer washes were used when signal was intense (> 100,000 cpm). The filters were blotted dry, covered with saran-wrap and arranged face-up in cassettes along with three sheets of X-ray film. The films were taped to each other so that they would not shift during the 0 to 8 day exposure in a -80°C freezer. Films were developed after three days to identify positive clones. Filters were re-used for sequential screening of different oligo-nucleotide repeats by stripping them between each use. Stripping consisted in washing the filters at room temperature in 100 mM NaOH, 10 mM EDTA, 0.1% SDS twice, followed by a 5× SSPE (1.2.1 175.3 g NaCl; 27.6 g NaHPO4-H20; 7.4 g EDTA; in 800 mL H20; adjust pH = 7.4 with aprox. 6.5 mL 10 N NaOH) rinse for 10 min.

### Clone identification and sequencing

Positive clones were identified by which filter they were on; which field within the filter they were in, and what address they had within the field. Clones could be identified by their position in the double-replicate 4 × 4 pattern found at each grid axis in the address system. Only double-spotted clones were selected. Any spots for which the replicate did not hybridize were considered false positives and were not selected. Putative simple sequence repeat-containing clones were picked from their appropriate position in the 384-well plate format glycerol stocks. DNA was extracted from an overnight culture by standard alkaline lysis procedures according to Sambrook [29] and used for sequencing initially from the 5' end using a SP6 primer and then from the 3' end with a T7 primer in cases where an SSR was not detected at the 5'end. Sequencing reactions were performed on ABI sequencers at Clemson University Genomics Institute and at CIAT. All sequences were searched for vector segments to check for insert integrity. The sequences containing a minimum of 100 high quality bases, with phred value of 20 or above, were trimmed to remove vector and adaptor sequences, blasted with blastn or blastx and an E-30 value threshold against the Genebank nr and EST databases and searched for simple sequence repeats as described below.

### Primer design and testing

SSRs were found in the sequenced clones using the SSR identification tool (SSRIT) available at http://www.gramene.org/ to screen for all possible repeats with sequences containing a minimum of five di-nucleotide, four tri-nucleotide or three tetra-nucleotide motif repeats used for primer design. Primer 3.0 http://www.genome.wi.mit.edu/ was then used to design primer pairs which would produce PCR amplification fragments that were on average 150 bp long. All PCR primers had consistent melting temperatures of 55°C or above, average lengths of 20 nucleotides or more and primer pairs were checked to make sure that they had similar melting temperatures and did not suffer from palindromes or end pairing. The newly-designed Microsatellites were tested against a standard survey of 18 genotypes from Blair et al. [13], among which a total of 7 were Andean (5 cultivated and 2 wild accessions) and 11 were Mesoamerican (10 cultivated and 1 wild accessions). The three wild accessions represented accessions from Argentina, Colombia and Mexico. Among the cultivated genotypes, 4 were Mesoamerican advanced breeding lines from CIAT (BAT477, BAT881, DOR364 and DOR390), one was a locally bred Andean large red seeded variety (Radical Cerinza) and the remainder were germplasm accessions (G lines) which were equally divided among the genepools. DNA for each of these genotypes was extracted by a mini prep method [30] and was quantified with a Hoefer DyNA Quant 2000 fluorometer for dilution to a standard concentration of 10 ng/ul before using in PCR reactions.

### PCR amplifications and allele detection

Standard PCR conditions from Blair et al. [13] were then used for amplification reactions: namely, a hot start of 92°C for 5 minutes; then 30 cycles of 92°C denaturing for 1 minute; 47°C annealing for 1 minute and 72°C extension for 2 minutes; followed by a 5 minute final extension at 72°C. The final volume for the PCR reaction was 20 μL which included 50 ng of genomic DNA, 0.1 mM of each of the forward and reverse primers, 10 mM Tris-HCl (pH 7.2), 50 mM KCl, 1.5 to 2.5 mM MgCl2, depending on the primer combination, 250 mM of total dNTP and 1 unit of Taq polymerase. After amplification, a volume of 5 μL of formamide, containing 0.4% bromophenol blue and 0.25% w/v xylene cyanol FF, was added to each PCR reaction and the mixture was denatured at 92°C for 2 minutes. Polymorphism was detected on 4% denaturing polyacrylamide (29:1 acrylamide: bis-acrylamide) gels that contained 5 M urea and 0.5× TBE and which were run in Sequi-Gen GT electrophoresis units (Biorad, Hercules, CA, USA) at a constant power of 120 W. Detection of PCR amplification products was via silver staining according to the Manufacturer's guide (Promega Inc., Madison, WI, USA) with some modifications, namely repeated use of the staining and developing solutions through a re-circulating tank system. Allele sizes were determined by comparison of SSR versus molecular weight standard band migration. A matrix of allele values and genotypes was used to calculate dissimilarity between genotypes and to construct a simple-matching neighbor joining dendogram which was subjected to 1000 bootstraps using the software program DARwin 5.0 [31].

## Results

The cDNA library used for this research consisted of 18,432 clones which were arrayed on filters of 36,864 spots each representing 48 plates of 384 clones for a total of 18,432 unique addresses. The preparation of the high-density filters was made possible by the high-throughput and automated nature of the robot used to pick colonies and array the final clones from the plate system to the filters. In addition, the arraying and screening of the library was facilitated by the high quality of the cDNA library itself which was reflected in all the clones (100%) having inserts, in the average insert size of 1.3 kb and in the transformation efficiency of 3.85 × 10^6^.

Hybridization of the cDNA library with SSR-containing probes detected a total of 1203 positive clones of which 886 were successfully sequenced. These represented 6.5 and 4.8% of the total number of clones in the library, respectively. The read length for the sequences was on average over 700 nucleotides. However, where sequences were short or of low quality it was usually because of difficulty in sequencing through simple sequence repeats from the 5 'end of the cDNA clones or through mononucleotide stretches, such as poly A tails encountered in sequencing from the 3' end. SSR loci were sequenced successfully and clearly identified for a total of 497 clones which represented 2.7% of the full cDNA library and 41.3% of the hybridizing clones. The frequency of individual SSR motifs found in the 5' and 3'sequencing is shown in Table 1.

**Table 1:** SSR repeats identified in the sequenced cDNA clones.

**SSR type**^1^	5' sequences	3' sequences	total	Percentage of total	Proportion 5' end
Di-nucleotides	181	28	209	42.1	86.6
ac/gt/ca/tg	44	4	48	9.7	91.7
ag/ct/ga/tc	124	13	137	27.6	90.5
at/ta	13	11	24	4.8	54.2
gc/cg	0	0	0	0.0	--
Tri-nucleotides	194	30	224	45.1	86.6
aag/aga/gaa/ttc/tct/ctt	24	1	25	5.0	96.0
aat/ata/taa/tta/tat/att	2	7	9	1.8	22.2
aac/aca/caa/ttg/tgt/gtt	34	2	36	7.2	94.4
acc/cac/cca/tgg/gtg/ggt	19	2	21	4.2	90.5
agc/cag/gca/tcg/gtc/cgt	68	15	83	16.7	81.9
agg/gag/gga/tcc/ctc/cct	30	2	32	6.4	93.8
atc/cat/tca/tag/gta/agt	4	1	5	1.0	80.0
ccg/gcc/cgc/ggc/cgg/gcg	8	0	8	1.6	100.0
gac/cga/acg/ctg/gct/tgc	5	0	5	1.0	100.0
Tetra-nucleotides	31	20	51	10.3	60.8
Penta-nucleotide	11	2	13	2.6	84.6

Total	417	80	497	100.0	-

^1^Complementary sequences for a given motif are given and were the basis for grouping of di-nucleotide and tri-nucleotide motif SSR.

In terms of repeat length, totals of 209 di-nucleotide and 224 tri-nucleotide motif SSRs were found in the successful sequences representing 42.1 and 45.1% of the total number of successfully sequenced clones, respectively with the remainder being tetra- or penta- nucleotide repeats similar to the di-nucleotide repeats we used as probes. Among the di-nucleotide repeats there were approximately three times more AG motif microsatellites (124) than AC motif microsatellites (48), and only a few AT microsatellites were found (24). Interestingly, while the AG and AC motif microsatellites were much more likely to be in the 5' sequences, about half of the AT microsatellites (11 out of 24) were found in the 3' sequences. Meanwhile, no CG microsatellites were found. Among the tri-nucleotide containing repeats, the most common by a factor of almost three was AGC with a total of 83 clones, followed by AAC and AGG with 36 and 31 clones, respectively. More than 20 clones each were found for AAG and ACC motifs; while less than 10 clones were found containing AAT or CCG motifs. The proportion of tri-nucleotide SSR at the 5' end was generally high, between 80 and 96%, except for the AAT motif for which it was 22%.

The most promising positive SSR containing clones were used to design microsatellite primers for the development of new markers for common bean (see Additional file 1). A total of 248 microsatellite primer pairs were designed from the positive cDNA clones after SSR probe hybridization. These targeted di-nucleotide or tri-nucleotide motifs in clones which had high-quality sequences flanking both sides of the repeat motif and good primer design sites in terms of GC content and non-annealing or non-palindromic sequences. We decided not to target tetra-nucleotide or penta-nucleotide repeat containing sequences since they usually are of low polymorphism. The new markers were named as part of the BMc series with this abbreviation standing for Bean Microsatellites based on cDNA clones and with a consecutive number starting at 121 and ending at 368 to distinguish from previous SSRs analyzed in Ramírez et al. [27].

**Figure 1:**
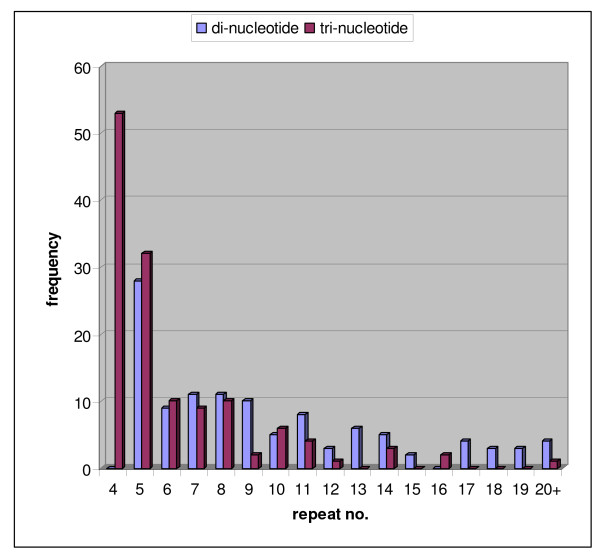
**Repeat numbers found in the di-nucleotide and tri-nucleotide motif simple sequence repeat loci**.

The newly-designed microsatellite markers were similar to the overall sequenced clones in terms of the SSR motifs they contained and the repeat length of the SSR themselves but concentrated on di-nucleotide and tri-nucleotide repeats since they generally have higher polymorphism than the longer motifs. Through blastn searches we showed that many loci were homologous to legume genes (84) or ESTs from common bean (210). The number of repeats for each of the repeat types is shown in Figure 1 with the average number being 9.59 repeats for the 112 di-nucleotide based markers and 6.18 repeats for the 136 tri-nucleotide based markers. The distribution of the repeat numbers for the markers showed skewing towards the lower end of the distribution with a few high repeat number microsatellites such as BMc367 which targeted a GA motif locus with 21 repeats or BMc256 which targeted an ATA motif locus with 39 repeats.

Most of the microsatellites targeted were simple rather than compound with a total of 203 microsatellites (81.6%) targeting a single motif and 45 microsatellites (18.4%) targeting more than one motif. Several of the microsatellites with a large number of total repeats detected compound SSRs, in the case of the di-nucleotides often this consisted of GA or CA motifs combined with AT motifs as was the case for BMc294 and BMc284. Meanwhile the high repeat number tri-nucleotide markers included compound SSR that had more than one repeat type such as BMc223 with the motif (CAG)10(GCA)6. A somewhat higher proportion (43/112) of dinucleotide markers fit the category of Class I microsatellites (10 or more repeats) compared to the proportion of trinucleotide markers (38/136) fitting this category (7 or more repeats).

**Figure 2:**
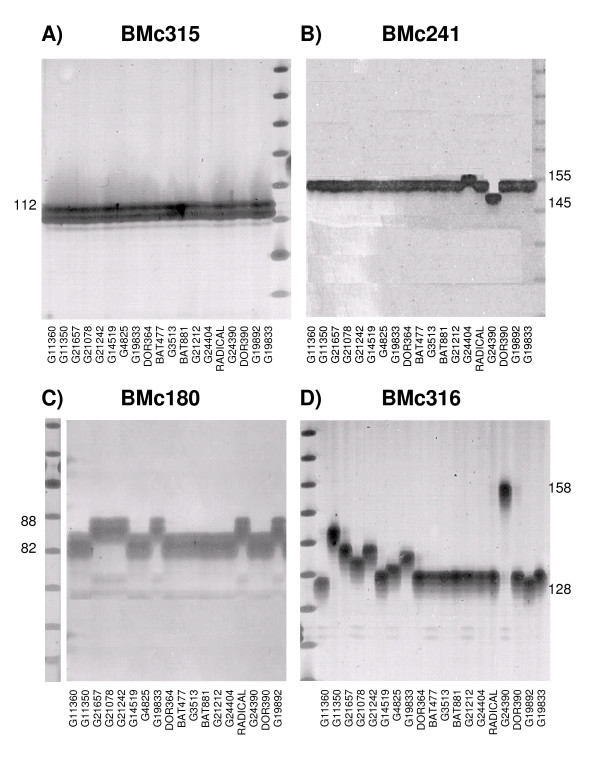
**Representative cDNA based microsatellite markers showing four patterns of diversity**. A) BMc315, a monomorphic microsatellite. B) BMc241, a polymorphic microsatellite that detected different alleles in the wild accessions, but not within the cultivated accessions. C) BMc180, a bi-allelic microsatellite that detected different alleles for Andean versus Mesoamerican accessions. D) BMc316, a more polymorphic microsatellite that detected different alleles across most of the accessions, especially those of the Andean genepool.

The diversity values for all the markers that amplified well were estimated based on the evaluation of 18 genotypes from the same mini-core panel evaluated by Blair et al. [13] that contained representative diversity from both genepools and from wild beans. The resulting marker evaluation could distinguish 1) monomorphic microsatellites that detected only one allele; 2) polymorphic microsatellites that detected different alleles in the wild accessions, but not within the cultivated accessions; 3) bi-allelic microsatellites that detected different alleles for Andean versus Mesoamerican accessions across various wild and cultivated genotypes and 4) more polymorphic microsatellites that detected different alleles across all or most of the accessions, especially those of the Andean genepool. Figure 2 shows the amplification pattern for each of these types of markers. A characteristic of the cDNA based microsatellites developed here was a low amount of stutter band and a sharp amplification product in the silver stained gels which made it easy to detect polymorphisms in the group of genotypes evaluated. Among the full set of markers, 122 primer pairs detected polymorphism across the full set of genotypes, while 82 markers were monomorphic due to conserved gene sequence. A total of 10 markers were polymorphic in the wild accessions but not in the cultivated accessions. The range in number of alleles detected in the germplasm survey was up to 15 alleles for the di-nucleotide repeat markers and up to 6 alleles for the tri-nucleotide markers with averages of 3.5 and 1.9, respectively.

**Figure 3:**
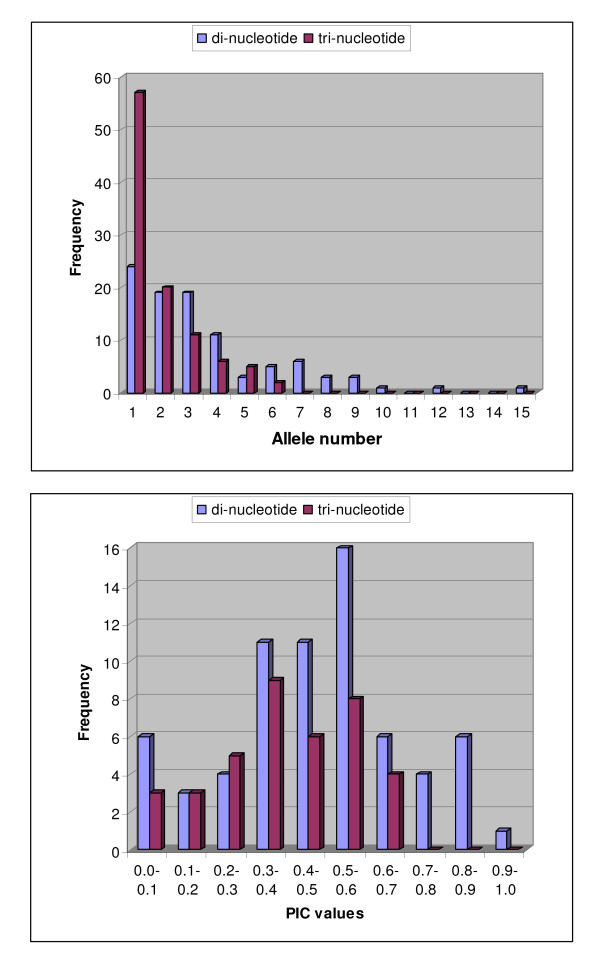
**Frequency distributions for A) allele number among all markers and B) PIC values among polymorphic markers for di-nucleotide and tri-nucleotide repeat motifs**.

Polymorphism information content (PIC) ranged from 0.000 to 0.9159, with the most polymorphic markers being BMc238, BMc292, BMc321, BMc171, BMc367, BMc294 and BMc226, all di-nucleotide repeat based markers with PIC values above 0.8000. The most polymorphic tri-nucleotide repeat based markers were BMc349, BMc229 and BMc335 with PIC values of 0.6171, 0.6268 and 0.6270, respectively. The average PIC value was higher for the di-nucleotide based markers (0.3544) than for the tri-nucleotide based markers (0.1563). The high PIC markers tended to be the SSRs with high repeat number and correlation between these values was highly significant (r = 0.575, P = 0.0000). Allele number was even more closely correlated with repeat number (r = 0.614, P = 0.000). The distributions of PIC values and allele numbers are shown in Figure 3. Average PIC values were 0.4795 and 0.3908 for the polymorphic di-nucleotide and tri-nucleotide based markers, respectively. Non-amplification was found for 17.7% of the markers and could have been due to either the forward or reverse primer not annealing to the genomic DNA due to intron sequences not considered when designing the exon-based primers or due to non-amplification with the PCR protocol used here which was designed for small fragments. Non-amplifying markers were not retested but are presented since they might amplify with touchdown PCR conditions or longer extension times.

**Figure 4:**
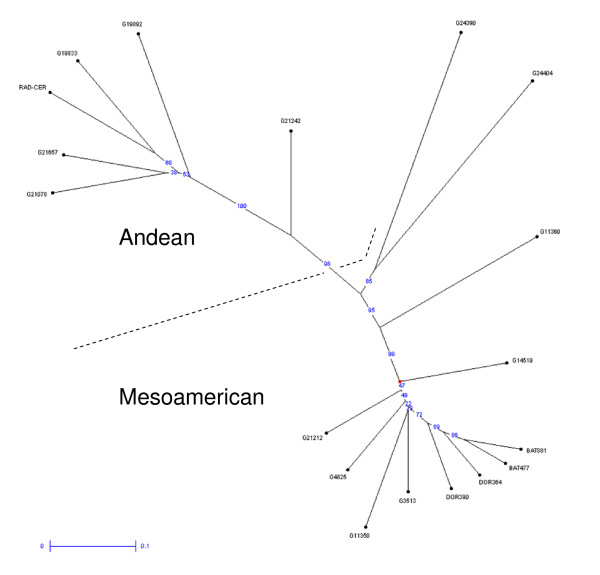
**Neighbor joining dendogram of relationships between wild (G19892, G24390 and G24404) and cultivated (all other) accessions of common bean based on 248 cDNA derived SSR markers with Andean and Mesoamerican genepools indicated**.

Among the accessions analyzed the most likely to be polymorphic using the new BMc markers were 1) the wild accession from Colombia (G24404); 2) the wild accession from Mexico (G24390); and 3) the race Peru accessions, G19833, G21657 or Radical Cerinza and 4) the Mesoamerican landraces G11350 or G14519. Generally, there were more polymorphisms within the Andean germplasm than within the Mesoamerican breeding lines or germplasm accessions. Figure 4 shows the genetic relationships between the genotypes and the separations of Andean and Mesoamerican genepools from each other and from the wild accessions G24390 and G24404 from Mexico and Colombia, respectively, which were supported by very high bootstrap values ranging from 95 to 100%. The Andean genepool included the Argentinean wild accession G19892 and the landrace G21242 was found to be intermediate between the genepools confirming results from Blair et al. [13].

## Discussion

The approach taken here for gene-based microsatellite development was based on the targeted identification of SSRs in high-density filter arrayed clones from a large cDNA library. This was facilitated by the robotic picking of a large number of clones (over 18 thousand) and the automated library duplication and filter preparation features of the Q-bot. Meanwhile, the cDNA library we used was made to represent a wide range of vegetative tissue from three week old, greenhouse-grown plants including young and old leaves, stem and vascular tissues, as well as vegetative meristems and therefore as a multi-tissue library was appropriate to upscale with this technology. To picture the scale of the library, a total of nearly 24 MB of non-redundant transcriptome sequences were represented in the filter set based on the number of clones screened, the low redundancy of clones and the average cDNA insert size of 1.3 kb, which compares favorably to other cDNA libraries that have been used in EST sequencing projects [27].

Hybridization of the cDNA library was straightforward in the filter format used. Positive hit addresses were confirmed through double replicate spots and the next product was a large number of SSR candidate loci which were then sequenced to confirm whether the hybridizing clones from each library had true SSR loci or were false positives. In this step, it was possible to discover the SSR motif and number of repetitions that each positive clone contained. It was important to sequence from both the 5' and 3' ends of the positive cDNA clones to obtain the full SSR and flanking sequences and to be able to design microsatellite primer pairs around the repeat motif. This was also important given the size of the cDNA clones, especially for the larger cDNA clones.

The use of di-nucleotide and tri-nucleotide SSR probes for filter hybridization and the sequencing confirmation of clones allowed us to calculate the frequencies of various types of motifs in the cDNA library. The results showed that the frequencies of di-nucleotide and tri-nucleotide clones were almost equal in the cDNA library although the tri-nucleotide positive clones were somewhat more frequent than the di-nucleotide positive clones. These results contrast with those of Yu et al. [14] who found 17 di-nucleotide and 11 tri-nucleotide SSRs for *Phaseolus *in their search of the NCBI gene database; and also with those of Hanai et al. [17] who found 137 di-nucleotide and 96 tri-nucleotide in their search of a unigene set from Melotto et al. [32]. Genomic microsatellites have tended to be more often based on di-nucleotide motifs than tri-nucleotide motifs in various un-enriched, small-insert libraries screened in our laboratory [33].

Our results suggest that tri-nucleotide repeat are more common in coding sequences than across the entire bean genome, which would be interesting given the triplet nature of tri-nucleotide SSR which is more compatible with the conserved nature of most open reading frames [2]. Tri-nucleotide repeats have usually been more common than di-nucleotide repeats in various EST collections although this is more pronounced in cereals than in dicotyledonous crops and less pronounced in full length or assembled ESTs [3,6,8,9]. Meanwhile, we were surprised to find that the cDNA clones we selected had a few tetra-nucleotide simple sequence repeats related to the motifs that were screened in the hybridization.

In terms of the frequency of individual motifs, the results indicate that GA motif microsatellites were more common than CA motif microsatellite in the sequences of the cDNA library used for this study with implications for the common bean transcriptome. The higher frequency found for GA motifs agrees with results of microsatellite development in several other plant species based on ESTs [3,9,34]. Another explanation for the higher prevalence of GA versus CA motif microsatellites in our study could be that the hybridization worked better for this probe although both oligo-nucleotides had the same melting temperature so this seems unlikely. Among the di-nucleotide motifs that were not used to screen the library, we also found a small number of AT-motif microsatellites, often in the same clones that contained GA or CA motif microsatellites. We pursued these markers since AT motif SSR have proven to be highly polymorphic in common bean [24]. Interestingly, the AT motifs were as likely to occur in the 3'end of the cDNA clones as in the 5'end of these. This was in contrast to the GA and CA microsatellites which were ten times more frequent in the 5'end than in the 3'end of the cDNA. Meanwhile, no CG motif microsatellites were found in the cDNA clones perhaps because we did not screen for this SSR type.

When comparing our results with those of Hanai et al. (2007) we can observe some similarities, as these authors found that AG motif SSR were four times more likely than AC motif microsatellites, that AT microsatellites were fairly common (27% of all dinucleotide SSR) and that no CG motif microsatellites were present in a set of 3,126 common bean unigenes. The differences between our study and theirs in terms of precise number of motifs may have been the result of the random nature of EST sequencing versus the hybridization approach we used, or because Hanai et al. [17] only searched 5' ESTs rather than both 5' and 3' end sequences as we did. It was significant that in both analyses, CG motif microsatellites were impossible to find.

Among the trinucleotide repeats, we found AGC microsatellites to be very common, representing 37.1% of the total for the tri-nucleotide SSR as might be expected since this was one of the motifs used as a hybridization probe. The next most common tri-nucleotide motifs (AAC and AGG) were also used as hybridization probes, but were less than half as frequent as this previous motif, representing little more than 5% of the total SSRs found. Two fairly common motifs not used as hybridization probes, AAG and ACC were also identified with the library screening procedure, perhaps because they were similar to the di-nucleotide motif probes used. Finally small number of other tri-nucleotide motif SSRs were found as compound repeats with the more common tri-nucleotide repeats or with the di-nucleotide repeats that had been hybridized with. One interesting trend for the ATA tri- nucleotide motif which was also used as a probe was that these SSR were more likely in the 3' end of the cDNA clones than in the 5' end just as we found for the AT di-nucleotide repeats. No other tri-nucleotide motif presented this same situation and in most cases very few of the tri-nucleotide motifs occurred in the 3' end which is similar to results from Thiel et al. [7] for barley where 5' and 3' ESTs yielded different amounts of polymorphic di-, tri and tetra-nucleotide repeats.

These results differ from the relative tri-nucleotide motif frequencies found by Yu et al. [14] or Hanai et al. [17] who identified larger numbers of ACC, AAG and ATC loci, perhaps because they did not use the hybridization approach we used and were instead screening unselected sequences. AAG tri-nucleotide motifs were common in soybean ESTs according to Gao et al. [3]. Finally in terms of the motifs found in this study, we did not screen the library for tetra-nucleotide motif microsatellites but also found them occasionally in the 3' end of the cDNA clones, while penta-nucleotide repeats were uncommon but located at the 5' end of the cDNA clones positive for this motif. Interestingly, Yu et al. [14] found a large number of tetra-nucleotide motifs in the sequences they searched, which averaged 1.35 kb per sequence, compared to Hanai et al. [17] who searched shorter EST sequences from Melotto et al. [32].

All together, these results give us a good idea of which motifs are common in cDNA sequences and at which end of the cDNAs they occur. The total percentage of ESTs that might be expected to contain SSRs is at least 6.52% based on the number of cDNA clones that hybridized with SSR probes in this study. If a larger number of hybridization probes of different motifs had been used, an even larger percentage of the cDNA clones would likely have been identified as containing SSR. The large size of the G19833 cDNA library and the comprehensiveness of the filter screening approach would make the discovery of further SSRs very likely and both library hybridization and EST sequencing as described in Ramírez et al. [27] will be useful for this effort. In other efforts to develop gene-based microsatellites from cDNA sequences, the frequency of SSRs has also been high. For example, in searching their unigene set, Hanai et al. [17] found that 7.01% of the genes contained SSR. Yu et al. [14] searching larger gene fragments as mentioned earlier, predicted that 23.8% of a limited set of entries from GenBank (206) contained SSR loci.

The source of the RNA for the cDNA library was made from G19833, a race Peru landrace from the Andean genepool, which is one parent of a genetic mapping population that has been used to map microsatellite markers [16,24], to study low P adaptation QTLs [35,36] and to create a BAC library to anchor to the first physical map for the species [37]. The screening of this library was complementary to our previous microsatellite marker development which was done with DOR364, a Mesoamerican source used to create un-enriched, small insert libraries [33] as well as marker development in Brazil which has used Perola and IAC-UNA, two other Mesoamerican varieties [17,21,22]. Other Andean sources used to clone microsatellites, such as Calima used for the enriched libraries of Gaitan et al. [18] have been from the Nueva Granada race rather than from race Peru. In comparison, G19833 is a valuable source material since it is the first genotype used to create a physical map for common bean [37] and the G19833 EST sequences have been compared to ESTs from Negro Jamapa 81, showing a large number of polymorphisms in contiged sequences [27].

The sequencing from both the 5'and 3'ends of positive cDNAs was especially valuable for discovering the full complement of microsatellites in the selected clones. This is in contrast to most other studies of gene-based microsatellites which in using the more commonly available 5' ESTs, may not give a complete picture of the frequency of microsatellites in expressed sequences, precisely because they rely on partial sequencing of the cDNA (ESTs). On the other hand, an advantage of EST mining of SSRs is the possibility of sampling genes represented in various cDNA libraries or genes expressed in different organs, at different developmental stages or under varying environmental conditions which can give a complete picture of transcribed genes [27]. The use of additional cDNA libraries, full length cDNA clones or 3' EST sequencing may be valuable for future microsatellite development, as will analysis with next generation sequencing techniques that can be used to shotgun transcriptomes and reconstruct them bioinformatically.

Another practical result of this study was that the careful analysis of both 5' and 3' end sequences allowed us to develop a large set of new gene-based microsatellites for diversity assessment or gene tagging projects in common bean. In terms of genetic diversity, the markers detected a moderate number of alleles compared to genomic microsatellites we have evaluated on the same set of genotypes [13,24], however the distribution of alleles was very predictive of genepool status and diversity was higher within the Andean compared to the Mesoamerican genepool showing some ascertainment bias as found before. It was notable that the most divergent accessions were the wild accessions from Colombia and Mexico and the new markers were very predictive of their relationship to the cultivated accessions based on high bootstrap values. The wild Argentinean accession meanwhile was found within the Andean genepool which agrees with results from Blair et al. [13] suggesting they are useful as gene-based markers for studying race structure and perhaps phylogeny if homoplasy is not a factor.

In addition to being valuable functional markers, the new microsatellites were highly transferable from cultivated to wild accessions. Testing of cDNA based microsatellites has shown them to also be transferable to tepary bean, *P. acutifolius*, which belongs to the tertiary genepool of common bean (this laboratory, unpublished). This suggests that the gene-based SSRs could be candidates for transfer beyond the boundaries of the tertiary genepool (to other *Phaseolus *species) or potentially to cowpea and other *Vigna *species. Supporting this hypothesis, some gene-derived microsatellites from common bean have been effective for diversity assessment in cowpea (S. Hearne, pers. Communication); and EST-derived SSRs are known to be better for transferability among related species than genomic SSR [6,38].

The overall success rate of marker development, from the step of sequencing to primer design was 57.3% for the di and tri- nucleotide markers which were targeted in our study. This is very similar to the success rate for SSR primer design from EST sequences from common bean (64.5%) reported by Hanai et al. [17]. In that study, 86 out of 240 ESTs had SSRs that were not flanked by unique sequences either because they were too close to the end of the sequences or had poor primer design sites, while in our study 185 out of 497 had such a situation. Careful design of microsatellite primers is important in obtaining a high percentage of amplification. Furthermore, having single copy PCR products between 90 and 250 bases in length allow an easy detection of microsatellite alleles on polyacrylamide gels, which makes these markers ideal for medium sized laboratories interested in genetic mapping.

## Conclusion

In summary, this study was successful at determining the relative microsatellite motif frequency in cDNA libraries based on a hybridization approach. In addition, the study produced a large set of Andean-derived, gene-based microsatellites for functional diversity analysis which are complementary to previously developed genomic and Mesoamerican derived markers. The markers were of moderate polymorphism but were very efficient at separating divergent wild and cultivated accessions as well as Andean and Mesoamerican genepools. Multiple microsatellite development approaches are important in common bean given the dual genepool nature of genetic diversity in the crop and the fact that ascertainment bias is a common problem with microsatellites. Gene-based markers from common bean are predicted to be useful for further phylogenetic analysis of the crop, for transfer to related species especially other cultivated *Phaseolus *species and for the development of a transcript map for common bean.

## Authors' contributions

MWB designed the study, analyzed data and wrote the paper. MM performed sequencing and primer design and analyzed microsatellite frequency, MCG conducted genotype polymorphism survey and FP hybridized and selected cDNA clones for sequencing. All authors read and approved the final version of the manuscript.

## Supplementary Material

Additional file 1 **Common bean EST-SSR markers from the BMc series**. Primer list, product size, blastn homology and GenBank entries for newly-developed SSR markers. Click here for file
