# Supplementary material for: Development and diversity of Andean-derived, gene-based microsatellites for common bean (Phaseolus vulgaris L.)
Source: BMC Plant Biol. 2009 Jul 31;9:100. doi: 10.1186/1471-2229-9-100 (PMC3091531; doi:10.1186/1471-2229-9-100)
Supplement: Additional file 1 — Common bean EST-SSR markers from the BMc series. Primer list, product size, blastn homology and GenBank entries for newly-developed SSR markers. [file 1471-2229-9-100-S1.doc]

**Additional File 1.** Primer sequences, annealing temperatures, expected product sizes, SSR motifs and blastn hits of 248 cDNA based microsatellites developed from the hybridization of SSR-containing motifs to a high density, common bean cDNA library filter.

| **Primer Name** | **GenBank Acc.** | **Motif** | **Exp. Size** | **Left Primer** | **Right primer** | **T_ann_** | **Blastn hit**  **(> E-30)** |
| --- | --- | --- | --- | --- | --- | --- | --- |
| BMc121 | GO477843 | (GA)19 | 160 | TGCATTCACCGCTATTACGA | CACTGTAGCCACCATGAGCA | 56 | NONE |
| BMc122 | GO477844 | (CT)6 | 128 | TCTCTCTTCACATAAACCCTAC | CTCTGCCATTGTGGATTG | 49 | dbj|AK285889.1 |
| BMc123 | GO477845 | (CT)8 | 164 | CCTTCCACCTTCTTCCCTTC | TTACCATTCATTGGTTTATTAGCA | 54 | NONE |
| BMc124 | GO477846 | (GA)12 | 122 | TGTCGGTTGTGAGACAGGAG | TTGGAGCTGCTACTCCCACT | 55 | ref|XM_002279954.1 |
| BMc125 | GO477847 | (CT)5 | 102 | GTTGCAATTCATCACCATGG | GCAGTGGGAGGGTATTTTG | 54 | gb|FJ654703.1 |
| BMc126 | GO477848 | (CT)8(TA)10 | 197 | CCCCTTTTCTCACCACTTTT | ATTGCTTGTGCGTGTTGTGT | 54 | NONE |
| BMc127 | GO477849 | (CT)5(CT)4 | 148 | CCCCACAAGGGCATATTTATC | TTGTGAGCAGGCCAGAGG | 56 | NONE |
| BMc128 | GO477850 | (TAA)5 | 181 | AATAACTGAGCAATAGAATGCCTAA | AAAGGGTCGATTGTGACTGTG | 54 | dbj|AK287017.1 |
| BMc129 | GO477851 | (GA)5(CT)7 | 211 | ACGTCCTCTACCAGCAAACA | TTTAGAACTGGGCACCTTGG | 54 | NONE |
| BMc130 | GO477852 | (AT)3(CTG)4(TCC)3 | 148 | CCTTGCCCACACCATACATC | ATGAGGAAGGAGAGGCTTTG | 55 | gb|AF402606.1| |
| BMc131 | GO477853 | (CA)8 | 172 | AAACTTTCTCTCTTGGGGAAGAA | CCTTTTGGCAACCTTGATGT | 55 | NONE |
| BMc132 | GO477854 | (GT)7 | 172 | GCTTACAACTTTACACACTCCTATG | GAAGCTGGTGGTGTTTTAATGG | 54 | gb|AC134322.25 |
| BMc133 | GO477855 | (CT)8(GA)6 | 122 | CCGGCCTGGCACTAAAAT | TCGATTTCAACTACTCATCATCG | 55 | gb|BT052346.1 |
| BMc134 | GO477856 | (CT)8(GA)6 | 144 | CTGCATCAGCAGCAACATTT | GCTCTCCTTGATGGACTTGG | 55 | gb|BT052346.1 |
| BMc135 | GO477857 | (CAT)4 | 120 | AACCATTTTTACCGATTTACCA | CCGAGTCCTAATGGGTTCC | 53 | dbj|AB304458.1 |
| BMc136 | GO477858 | (CA)7 | 126 | GTCAGTAGCAACAACTGTCAAA | GCGAGAAACCACAGTGACCT | 53 | dbj|AK245839.1 |
| BMc137 | GO477859 | (CT)11 | 143 | TTATGGTGGAACAAGCTCCTG | GAAGACTGAATTGGTGAACCTAGAA | 55 | NONE |
| BMc138 | GO477860 | (CCA)7 | 120 | CCAAATCTGATTCATACGAACA | GGGAGGGACATGGACTTTCT | 54 | NONE |
| BMc139 | GO477861 | (GCT)6 | 124 | TGAGTCGACATCAAGCCAGA | ACCGGAGGGTGAAGAGTGTA | 55 | dbj|AP004528.1 |
| BMc140 | GO477862 | (AG)13 | 160 | TGCATTCACCGCTATTACCA | CAATGTAGCCACCATGAGCA | 55 | NONE |
| BMc141 | GO477863 | (CA)5 | 125 | TCTCTCTTCACATAAACCCTACAAA | TGTCATTGTGGATTGGGAGA | 54 | NONE |
| BMc142 | GO477864 | (TGC)5 | 121 | TTGGAACTGGTTCTGAGTTGG | TCATGTGACTGCTGATCAAAAA | 55 | gb|M75856.1 |
| BMc143 | GO477865 | (TGC)5 | 128 | GGGTGGCTACACCATTTCAG | TCACAGCTTTTCTTTCGTTCC | 55 | gb|AY007525.1 |
| BMc144 | GO477866 | (CCG)4 | 106 | TCGCTAGTCACGCTCAGAAG | GAAGATTCCATCACCGTATCG | 54 | dbj|AK243916.1 |
| BMc145 | GO477867 | (CA)5 | 139 | CCCCAACACGGAATAGAAAC | TGGTTGACATGCTTTGCATC | 55 | NONE |
| BMc146 | GO477868 | (CCT)5 | 113 | TCCTCCTTTCCCTCCTCCTA | GATGTAGACATGGGCGACAA | 55 | NONE |
| BMc147 | GO477869 | (TC)5 | 146 | CTTCACCCGTGTTTCATTCTC | ATTCGTTCCACCAGGCAGT | 55 | NONE |
| BMc148 | GO477870 | (GCA)5 | 103 | GCCATGCAACCAGGATTCTA | TACCAAATTGCAAGGGCATT | 56 | NONE |
| BMc149 | GO477871 | (CA)5(GCT)6 | 96 | CCCACTCATCAAATTACTCAACC | TGCATCTGCACTGATTTGGT | 55 | dbj|AK285415.1 |
| BMc150 | GO477872 | (CAG)5 | 107 | TGAAGTGAAAGAGAAACGACGA | TCCGCAACATGTTTGATCTC | 55 | dbj|AK244090.1 |
| BMc151 | GO477873 | (TGC)5 | 111 | CCACCGACAACAAGGAAGAA | CTGTCGCTTCTGCTACGTCA | 56 | NONE |
| BMc152 | GO477874 | (TCG)5 | 101 | CAGACAGTACCACCCGACAA | GCGCCAGGAACGAGTATAGA | 55 | NONE |
| BMc153 | GO477875 | (CT)5(CAG)4 | 100 | TGGAAGTCCCTTGTTCCTGT | TGGCTTGTTCAAGCTGAGG | 55 | dbj|AK286124.1 |
| BMc154 | GO477876 | (CTT)4 | 91 | CACAACGCCTACCTCTGCTC | AGGCCTCACAGACCCAGAC | 56 | NONE |
| BMc155 | GO477877 | (ACA)4 | 99 | TCTTGGAGGCTTCAACAAGG | TTGAGGTTGCTGTTGAGGTG | 55 | NONE |
| BMc156 | GO477878 | (CAA)6 | 87 | TTCACACACTCTCCCCCTTC | TTTCTTGCCCTAGTGGTGCT | 55 | NONE |
| BMc157 | GO477879 | (GT)6 | 148 | GAGGCAACTGACGGTAGCA | TCGCTCAGAAGTAGGTTTTCG | 55 | gb|AC144657.6 |
| BMc158 | GO477880 | (CTC)7 | 114 | CCCTTCCAAGAACCTCAAGA | CTCAAATCATCCCCAGTTGC | 55 | NONE |
| BMc159 | GO477881 | (CAG)8 | 95 | GTACCAGCAACAACCGCATT | CATTGGCGTCTCTTCTCAGC | 54 | dbj|AK244139.1 |
| BMc160 | GO477882 | (CCT)4 | 159 | AGGTACCGGAGTGGATGAAT | GAGAGGCATTGTGATTAGTTGG | 52 | NONE |
| BMc161 | GO477883 | (TC)9 | 129 | CCGCTCTTAACCTGTCACCT | ACCGTGTATTTGAGCGGTTG | 54 | dbj|AK244872.1 |
| BMc162 | na | (GT)6 | 107 | TGGGATATCTCTCTGGATGAAA | AGATTATCTGTTCGCTCAGAAG | 51 | gb|AF322116.2| |
| BMc163 | na | (CTC)7 | 114 | CCCTTCCAAGAACCTCAAGA | CTCAAATCATCCCCAGTTGC | 53 | dbj|AK285929.1 |
| BMc164 | GO477884 | (GCT)5(AAGG)3 | 157 | TTGGAACTGGTTCTGAGTTGG | GCTGTTACAGAAGCAGGGAGA | 53 | gb|EU196765.1 |
| BMc165 | GO477885 | (CA)5(CT)5(GAG)4 | 80 | GCCGCACACATACACTCTCA | CGCTCTGCCATTGTGGAT | 54 | gb|DQ503751.1 |
| BMc166 | GO477886 | (TCC)4 | 131 | GACTGTGTCATCTCCGACGA | AACTGGCCTAGGGTTTGGAT | 53 | dbj|AK244017.1 |
| BMc167 | GO477887 | (TCC)4 | 121 | CACGGAGAAGGACCTATTCG | GGAAGAGGTGGAAGATGACG | 53 | ref|XM_002326449.1 |
| BMc168 | GO477888 | (CGA)7 | 172 | CTCTCACCGACACAAACACC | GACGTCGTTTTCGTCGTAGC | 53 | NONE |
| BMc169 | GO477889 | (AG)5 | 104 | CTACTCCATGGGGCTTCTCA | TTCAATTCCCATGGTCTGAA | 53 | gb|AF228501.1| |
| BMc170 | GO477890 | (TTC)4 | 103 | ACATTCTTGGGCAGCCTCTA | AGTTGGCAAGGTTGGAGATG | 54 | NONE |
| BMc171 | GO477891 | (CT)11 | 121 | CCTTTCACTTCACTTGTGGTTC | GCCATGGCTGATTCAGTAGC | 54 | dbj|AK244017.1 |
| BMc172 | GO477892 | (CA)4(CACAA)3 | 126 | AGTTTCGGAGGGAACACACA | TGGTGTTCTCTGGGTTTGGT | 55 | gb|AC148405.5 |
| BMc173 | GO477893 | (TCG)4 | 101 | AGAAGCTCCATGCCAAAGG | GAAACCGACGGATCTAAGAGG | 54 | NONE |
| BMc174 | GO477894 | (AG)6 | 117 | CACACGCACACGTTGTTCTA | TGGCCCTTCTTTCTCTCAAC | 54 | NONE |
| BMc175 | GO477895 | (CCT)4 | 117 | CACCATCCACCGTCTTCTG | AGGCCTCACAGACCCAGAC | 54 | NONE |
| BMc176 | GO477896 | (GA)5(AG)8 | 130 | AGACACAACAACATTAGAGAACAGA | ATCCCGAAATGCAATGGTAA | 52 | dbj|AK285873.1 |
| BMc177 | GO477897 | (CCT)4 | 148 | TCCCACCACACTCCTTCTAA | CGTCAGCTTGCTGGAAGTTT | 53 | NONE |
| BMc178 | GO477898 | (CGC)4 | 104 | GAGGAGCTTGGTTTGCTCAC | GAGGTGGTGGTGATGGTGAT | 54 | ref|XM_002281256.1 |
| BMc179 | GO477899 | (CGC)5 | 93 | TGTCGGAGTTGTTGGTCTCA | TGCCTAATGGACCTCTTGGA | 54 | dbj|AK286732.1 |
| BMc180 | GO477900 | (GCT)6 | 113 | CACTTCTGTTGGGACCAATG | AAACCACTTTGAACTTTCAC | 49 | NONE |
| BMc181 | GO477901 | (CT)18 | 126 | CTTTTCTCCTTTCTCTCTCCGG | GTTTTGTGAGCCATGGAGGA | 55 | NONE |
| BMc182 | GO477902 | (GAA)4 | 107 | CCATTCTCAGGGAGCAAGAG | GGACACAGTTTGCCATTCGT | 54 | NONE |
| BMc183 | GO477903 | (CGA)4 | 123 | CTCCACCTCCCTCTTCTACG | AGAGTGGAGAGGTCGAGAGC | 52 | dbj|AK285440.1 |
| BMc184 | GO477904 | (ATC)7 | 136 | GCAGTTCGATTAACGGAGAG | GCCCATATGTGTGGAGTTGA | 52 | gb|AC235400.1 |
| BMc185 | GO477905 | (GAA)5 | 131 | GGAGTTGTTCAAGGGTCAGG | AATTCGAATCCGCAGCAG | 53 | NONE |
| BMc186 | GO477906 | (CTG)4 | 95 | CCGCCTAACAAACAACATCA | CAGAAACAAGCATCCAACAGG | 54 | gb|EF571299.1 |
| BMc187 | GO477907 | (GAG)8 | 122 | GAGCAAGAGTCCTCATCACG | GTGGGCTCGTTCTCGTTG | 53 | gb|AC235190.1 |
| BMc188 | GO477908 | (AGC)6 | 138 | CAGCGCGACACTAATTTGG | GATTCGGTTCATCCTCTCCA | 55 | gb|DQ503767.1 |
| BMc189 | GO477909 | (CCG)4 | 93 | TCGCTAGTCACGCTCAGAAG | CCGTATCGGTAGTGATGTCG | 53 | gb|AC146548.12 |
| BMc190 | GO477910 | (TC)6(TGTGT)3 | 108 | GCGAGACCCGTTGTGTGT | GGGAGCGAAACAGAGAGAGA | 54 | gb|AF139464.2| |
| BMc191 | GO477911 | (CAC)4 | 105 | CTGTCTCTTGCATAGCACGG | GCTGTGTTGCTCGTGGTTT | 53 | NONE |
| BMc192 | GO477912 | (TC)9 | 101 | GCAGGGAGAAAAATGGATTG | TCCGTGTCTTCCATAGCAAA | 53 | NONE |
| BMc193 | GO477913 | (CCT)4 | 96 | ACATCATCCCCATGGACACT | CGGCCTCGTCGTAGTAGAAT | 53 | NONE |
| BMc194 | GO477914 | (AG)5 | 120 | CCGCGTTTAGGTTGCTCTAA | TCCCATGGTCTGAACAGAAA | 54 | dbj|AK285440.1 |
| BMc195 | GO477915 | (TG)6 | 103 | GAAGAAATCGTCGTTGTTGTGA | GTGGGAAGCATCCCTGATTT | 55 | dbj|AK285440.1 |
| BMc196 | GO477916 | (ACG)4 | 89 | GTCGATAATGCCACCGAAAG | TTAGCCTCTTCATCGGGAAC | 54 | NONE |
| BMc197 | GO477917 | (TC)8 | 128 | CCATTCGCACCTTTGACTCT | CCATATCACAGACACGCCTTT | 54 | NONE |
| BMc198 | GO477918 | (CCT)4 | 88 | TTGTTACCGAAAGCCCACAT | TCATTCTGTTCTTCGCCTGA | 55 | dbj|AK245131.1 |
| BMc199 | GO477919 | (TC)11 | 90 | CGTCCGACAACATCCAAAT | CAGCGCATACTTCTTTTCTC | 51 | NONE |
| BMc200 | GO477920 | (AC)5 | 111 | CCCATATCCTTCTTCAGCAA | GGTCTGAGGGAGTGAGTGG | 50 | gb|DQ857273.1 |
| BMc201 | GO477921 | (CA)7 | 108 | AAGCAGTGGTTGGCCAATTT | TTTCCAATCCAATGTGCAAC | 54 | gb|AC159536.21 |
| BMc202 | GO477922 | (AG)8 | 94 | AGCTCCAGAGCAGTGCCTAA | GCGAAATGGCTTCTTCACTC | 54 | dbj|AK244017.1 |
| BMc203 | GO477923 | (GGA)4 | 141 | ACTCCAACAAATGCAGCAAA | TTAAGCGCCTGGTGGTTCT | 53 | gb|DQ857254.1 |
| BMc204 | GO477924 | (AC)7 | 101 | CCCTTGTGATGATTCCATCTG | TGATAAGTTGGTGTGTGAGACTAGA | 53 | gb|AF228501.1| |
| BMc205 | GO477925 | (CCA)4 | 153 | ACCCCTCCACCATGTTCCT | CCCAGGGGGAGAGGTAGTT | 55 | gb|AF014399.1| |
| BMc206 | GO477926 | (TC)8 | 100 | CTCTCATCCATTCGCACCTT | ACCCCACTTGGTCTTCGTCT | 55 | NONE |
| BMc207 | GO477927 | (TC)5 | 92 | CCCTATAATCCACGCCTCCT | GGGAACAGAAAGGTAAAGGC | 53 | NONE |
| BMc208 | GO477928 | (TC)6 | 103 | CGACACAGCATTCGCTACAC | ATGAATTCAGCGAGGAAAGG | 54 | gb|DQ503759.1 |
| BMc209 | GO477929 | (AAG)4(TC)5 | 142 | CCCGGAAATAGAAACCCTTT | AGTGGTCTTGGTGGGAATGT | 53 | NONE |
| BMc210 | GO477930 | (CAG)5 | 103 | CCAGCTGCAACAACAGCA | GTCAGCGACAGCTCCGTAAT | 55 | NONE |
| BMc211 | GO477931 | (TCT)4 | 108 | TCACTGGATTCAAGCTGGTTT | GAAAGTGCACGTGGGAGAGT | 54 | NONE |
| BMc212 | GO477932 | (TC)8(TG)6 | 199 | CCCGCTCTAAATAATTCTTCATTCC | GCAAGCAGTCCCTTGGTAGA | 56 | NONE |
| BMc213 | GO477933 | (CA)5 | 102 | ATTACTCGGGCACCATGTTC | ATGGTGGTTGACATGCTTTG | 53 | dbj|AK285415.1 |
| BMc214 | GO477934 | (CA)5(AC)6(CTT)6 | 148 | TTCTGATATTTCCTCCACCACA | TGGTTTGTGTTTGGGAATGA | 53 | NONE |
| BMc215 | GO477935 | (GAC)6 | 98 | TAAGACGTGAAGGACGAAGC | GAACCCTCAGCACCACCTT | 52 | gb|DQ787027.1 |
| BMc216 | GO477936 | (CAA)6 | 96 | GGCTTTCATCCCATACTACACA | TGGTGCTTGCACTCATTCTC | 53 | NONE |
| BMc217 | GO477937 | (CT)6 | 117 | CACCGCAACAGAATATAAAACAAC | AAGGATCGTGACGCCATAGA | 54 | NONE |
| BMc218 | GO477938 | (TC)5 | 152 | ACTTCACCCGTGTTTCATTC | CCTTGATTCGTTCCACCAG | 52 | NONE |
| BMc219 | GO477939 | (AG)5 | 94 | TTCTCTTGGTCGGGTTTCCT | AGCTTGAGTTCTGCAGCGTAG | 55 | NONE |
| BMc220 | GO477940 | (TC)10 | 112 | AAGCAACCAACAACAACAAC | CGCACATCAACAGCGCATA | 53 | gb|DQ503761.1 |
| BMc221 | GO477941 | (TG)8 | 99 | CTGAGTTTTGAGCGTGTTTTT | GAGGTCCACTTTTTCCACCA | 52 | NONE |
| BMc222 | GO477942 | (CA)9 | 143 | AAACTCCAACGCCGTTCTCT | CTCCGAAAATGTAGGGATTCA | 54 | NONE |
| BMc223 | GO477943 | (CAG)10(GCA)6 | 157 | AGCTTCCTCAGGTCCAACAA | TGAGGCTGCAGTTGCTGTAA | 54 | NONE |
| BMc224 | GO477944 | (TC)10 | 116 | CCACTTTATAATTTCTACTACTTCTCTC | GGAGCTTAAGAAGGTCAATTC | 49 | NONE |
| BMc225 | GO477945 | (TG)8 | 129 | CATCTTTACTGAGCAATCGAG | GCCAAGAACTGAATCCGAAG | 51 | dbj|AK285895.1 |
| BMc226 | GO477946 | (CT)14(TC)5 | 89 | GAAAACCCTCTCTCTCTCTCTCT | TCAGAATGGTTGCAGGAGTG | 52 | NONE |
| BMc227 | GO477947 | (TCC)8 | 100 | TCACGCGCTTTATCTCTTCC | AATGACCTGGGGGATGGTAG | 55 | dbj|AB372848.1 |
| BMc228 | GO477948 | (AC)5 | 114 | CACCCTCTCATCTTTCAAACC | CATCTCAACAGTCCATGCAA | 52 | gb|BT051874.1 |
| BMc229 | GO477949 | (TTC)5 | 98 | TTCCTCTCTCACTCTCATCAACA | CCAAAACCAAAACCAAAAGC | 53 | dbj|AK286485.1 |
| BMc230 | GO477950 | (CAA)5 | 104 | CAAGAGGCCTGATCAGAGGA | TGTTCCACCCATCATTGAAA | 54 | dbj|AK285445.1 |
| BMc231 | GO477951 | (TA)9 | 147 | TTTTGGTCGTGATGTCTCCA | ATAAGTTAAACCACCCACAGGT | 52 | gb|DQ822918.1 |
| BMc232 | GO477952 | (CAA)5 | 82 | GACAGTAACAAAGCCAAACATCA | GGGAGAACAACACCAGAAGC | 53 | NONE |
| BMc233 | GO477953 | (GGT)5 | 99 | AGGGGAAGGATATTGTTGAGG | CAGACGCAGCAGCAACAG | 53 | NONE |
| BMc234 | GO477954 | (TC)6(CT)11 | 93 | CAACACCATAACCAAAACGACA | ACGGAGGAAGGAGATGGAGT | 54 | NONE |
| BMc235 | GO477955 | (TGC)4 | 135 | CACCAAGAAGTGTGCCAGAG | CATTTTGACGAGGCGTGTTT | 54 | NONE |
| BMc236 | GO477956 | (CTG)4 | 98 | CTTCGGGTCCTGGTTGTTTA | CAATTTGTGCAACCCAAGGT | 55 | dbj|AK245286.1 |
| BMc237 | GO477957 | (CT)10 | 136 | CATCATCTTTCATCTGCACCA | TGATCTTGGCACTCAAGCAG | 54 | NONE |
| BMc238 | GO477958 | (CT)20(GAC)3 | 142 | CCAAACCTCTCTCTCTCTCTCTC | CGAACTCTTCGTCCTCCTGA | 52 | NONE |
| BMc239 | GO477959 | (GAC)5 | 96 | CTTTCTATGGATGGCATCTCA | *TCGAACTCTTCGTCCTCCTG* | 53 | NONE |
| BMc240 | GO477960 | (TC)12 | 96 | CGCGTCCGTTTCATTCTATT | AGAGAAGCGCCCATTGTTT | 54 | gb|AY830931.1 |
| BMc241 | GO477961 | (TC)7 | 147 | TGTGATCTGAATCTATTCCATTCC | TGTCTGAGGATGAGCTTGGA | 53 | dbj|AK285445.1 |
| BMc242 | GO477962 | (CAA)4 | 91 | TCATCAAAGGGCGTTGCTC | CATCATTTCCGCTTGATGCT | 56 | dbj|AP010552.1 |
| BMc243 | GO477963 | (ATC)6 | 87 | CCCACTCTCCCTTTCTCTCAC | GGGAAGAAGGAGGGTCAAAA | 55 | gb|DQ822976.1 |
| BMc244 | GO477964 | (AT)5 | 178 | CGACTCTTACTACTAATTCATTTTTG | AAGCATTATGAAATCTCCAAGA | 50 | dbj|AK244851.1 |
| BMc245 | GO477965 | (TCA)7 | 102 | ACTTGGTTCTGCACTGGATC | AGCCCCTCTTGGAGGCAA | 54 | emb|AJ867393.1 |
| BMc246 | GO477966 | (TC)5 | 111 | TGCCTCGAAATTAGGATTGG | TGCAAATTCTGGGCGAGTAG | 54 | NONE |
| BMc247 | GO477967 | (TCG)5(TCC)6 | 128 | TGCAATCGCAACAACAAATC | GCATAAGTCCAGTCCCCAGA | 55 | NONE |
| BMc248 | GO477968 | (TC)14 | 90 | CAAGCAAAATCTCTCCTTTTC | ACCCTAGTGGGTGAGTGTGG | 51 | NONE |
| BMc249 | GO477969 | (CGA)4(GAC)4 | 86 | ACCACTCCCACGTCGAAATA | CAAGAGCCAGGAAGCGGTT | 56 | gb|BT051874.1 |
| BMc250 | GO477970 | (CA)5 | 105 | TTGCATTCTCTTCCCACTCA | CATGGAAGGCTTTCACCAAG | 55 | gb|DQ224370.1 |
| BMc251 | GO477971 | (CGG)5 | 104 | CAGTTCAAGGAGGACGTTTG | ACAGAGACATTAACCCCAGCTT | 52 | NONE |
| BMc252 | GO477972 | (AGC)4 | 96 | CAGAAGCGCAAAAGTCCAG | TTTCCTTGGAGGAGGTGAAG | 53 | NONE |
| BMc253 | GO477973 | (CAG)5 | 91 | GCGATCTCAGTCAACAGCAG | TGCATAGCCATCCATTGGT | 53 | dbj|AP004926.1 |
| BMc254 | GO477974 | (AC)5 | 136 | TCAACTCACACACCTTCTCCA | GGGCCACAGATGAAAGTTGA | 54 | emb|AM476036.1 |
| BMc255 | GO477975 | (AT)5 | 96 | TCCGACATGCATAAACACAGT | GTCGTGTGTCTGCAATGAA | 53 | gb|AC235304.1 |
| BMc256 | GO477976 | (AAT)39 | 211 | GTTTGAACCGGTCCACGTT | GCAATCATTGTGCTCCTCATT | 55 | NONE |
| BMc257 | GO477977 | (TGC)4 | 118 | CCTATGATCAACAACAACCTTT | GATGTTGGCCGAATTGAAA | 53 | NONE |
| BMc258 | GO477978 | (TC)5 | 164 | TCATCATATCCCACATATCTACACC | CAGGTGCGACATCTTCACAT | 54 | NONE |
| BMc259 | GO477979 | (ATA)10 | 149 | TCACCTCTCATCATCTCATTCA | CCTTTTAGTTATTATGAGGTGGTGGT | 54 | emb|AJ867398.1 |
| BMc260 | GO477980 | (TTCC)3(CAG)5 | 186 | ATGTTGATCCTGCTTCAATGC | TTGGAACTGGTTCTGAGTTGG | 55 | gb|EF147793.1 |
| BMc261 | GO477981 | (TA)5 | 141 | CACACGAGCAGCTATTTCATA | GGATAACGGCAATAGGTTCTTG | 53 | NONE |
| BMc262 | GO477982 | (TA)5 | 136 | GCTACACACCAGCAGCTATTTC | CAAGAGGTTCTTGCCTTAAGTT | 53 | NONE |
| BMc263 | GO477983 | (TTC)4 | 80 | TGTTCGTGATCACAGTCTGTTCTT | AGTTTGGAATGTAAAGTGTCCTCTG | 56 | NONE |
| BMc264 | GO477984 | (AAT)10 | 133 | TCCATTCTCTCACCACCAAA | GGGATTCGAAGTGATTCTCC | 54 | NONE |
| BMc265 | GO477985 | (GT)7 | 109 | CACAGCTTACAACTTTACACACTCC | ATTTGGCCGATAACATCTGA | 54 | gb|AC134322.25 |
| BMc266 | GO477986 | (CAG)5 | 120 | TTCATCTGACTGCTCATCAA | GGAACTGGTTCTGAGTTGGAA | 52 | gb|EF147793.1 |
| BMc267 | GO477987 | (ACC)6(TTC)4(CAC)4 | 170 | GGACCCACCATCACCATAA | GTGGTGGATACAGCCGTGA | 55 | gb|AC119419.10 |
| BMc268 | na | (CAC)5 | 129 | TCCTCTTTCTTTGCCTCAGC | GGTTATTGCTGCTGGTAGGG | 55 | dbj|AK245314.1 |
| BMc269 | GO477988 | (CTT)10 | 91 | TGCCTTCATCATTGCCACTA | AGAATTGAACGGAGTAATGGTC | 54 | NONE |
| BMc270 | GO477989 | (CGT)4(TCG)5 | 166 | AGACGCCTAACCCTTCCAAT | GCGAGTGGTCGTGTTTCAG | 55 | NONE |
| BMc271 | GO477990 | (GTA)5 | 142 | TGATGGTTGTGTTTCTCAGTAGTTG | TGCCAACAAATGGAAGTGAT | 55 | dbj|AK286485.1 |
| BMc272 | GO477991 | (TC)18 | 213 | CATTCAATGCGGGAATTTTG | CCAGTGGGTTCGTTTACATTG | 56 | NONE |
| BMc273 | GO477992 | (CAG)4(CAA)4(GCA)6 | 94 | TTCAAGACCACAGCAACTGG | GGGATTCTGCTGTTGAATGAG | 55 | gb|DQ503714.1 |
| BMc274 | GO477993 | (CAG)5 | 120 | GGTAATCACATGCTCAACAAATC | TTGTCCAAGTCCCATCATCA | 54 | NONE |
| BMc275 | GO477994 | (CT)7 | 141 | AACTCTCTCTCTCTCTTTCTCCA | CCACAACACTCCAAATCCAA | 53 | NONE |
| BMc276 | GO477995 | (TC)9 | 116 | CCTTAACCATCAACCTTTCTCTCT | ACTACGGGATTGGTTTGCAT | 54 | NONE |
| BMc277 | GO477996 | (CCT)4 | 91 | CACAACGCCTACCTCTGCTC | AGGCCTCACAGACCCAGAC | 56 | gb|DQ371898.1 |
| BMc278 | GO477997 | (GAC)4 | 91 | ACCACTCCCACGTCGAAATA | TTGAGCAAGAGCCAGGAAG | 55 | NONE |
| BMc279 | GO477998 | (CAG)5 | 131 | GATGATGTCCCCACATTCAT | CCAGATGGTCCTGATCTCGT | 54 | NONE |
| BMc280 | GO477999 | (AG)17 | 129 | GAGGAAATATTGGTGGCAGAAA | CGCAATAAGGAGGTTGAGGA | 55 | gb|EU112625.1 |
| BMc281 | GO478000 | (CA)5 | 82 | ACGCGTGGGTAAAACATCAAA | AGACCAGACGAACCGGAAAG | 57 | NONE |
| BMc282 | GO478001 | (AT)5 | 121 | CAAGGATCAAAGGCAGGTTC | AGAAGCAGCAGCATCATACA | 53 | gb|DQ503716.1 |
| BMc283 | GO478002 | (TC)12 | 83 | CAAAGTCCCACTCTTCTCTCTC | TCAGCAAACCCTAATTGGAA | 53 | gb|AC140551.8 |
| BMc284 | GO478003 | (CA)12(TA)15 | 189 | CCCTATTTATCTGCAAATTCACGAG | GGGAGTGTAGATCAAGAGATTAAG | 54 | emb|AJ867393.1 |
| BMc285 | GO478004 | (CT)11 | 136 | GGACAATGGGACCTGGTATG | GATGCAAATGAGAAGCTGCA | 55 | emb|AJ867393.1 |
| BMc286 | GO478005 | (CAG)7 | 93 | CAACGGCGATCTCAGTCAG | ATAGCCATCCATTGGTGCTG | 56 | dbj|AP004926.1 |
| BMc287 | GO478006 | (CCA)5 | 116 | TGCCCTATGTTCACCCTCAT | CCAGATGGTCCTGATCTCGT | 55 | NONE |
| BMc288 | GO478007 | (CT)6 | 135 | CAATCACAACCCTTTTCTCCT | TTTGTGAGCCATGGAGGAAG | 55 | NONE |
| BMc289 | GO478008 | (TGG)4 | 185 | CTGTATTGTCAGAACGCTGTC | GGAGCCATCAGATGACCACT | 53 | dbj|AK285215.1 |
| BMc290 | GO478009 | (TC)5 | 110 | TCGACCCTCAAATCCAAATC | AGCGGAGAGTTTGAAAGCTGA | 56 | NONE |
| BMc291 | GO478010 | (GAC)6 | 98 | GACGCTCCCGTTGTATAAGA | CCTTGAGCACCGTCTTCCT | 54 | gb|DQ503715.1 |
| BMc292 | GO478011 | (TC)6(TC)4 | 290 | CCTTATTATACTTGTGGTCTCTCTCTC | CGAGGATGAACAGAGCATGA | 54 | NONE |
| BMc293 | GO478012 | (CAC)4 | 92 | CACTGATCCATCAACCCAAA | GGGTGGAGTTGGAAAGTGAG | 54 | NONE |
| BMc294 | GO478013 | (AT)7(AT)10(TG)6 | 123 | GGTCGTGATGTCTCCATTT | TACCTCCCATCATGCACTTAC | 52 | gb|EU112620.1 |
| BMc295 | GO478014 | (CAA)5(TC)5 | 114 | CCACGCGTGCGCAAC | CACATCAACAGCGCATACCT | 57 | dbj|AK287017.1 |
| BMc296 | GO478015 | (TA)8 | 123 | TGGTCGTGATGTCTCCATTT | CCTCCCATCAGGAACTTTGA | 54 | NONE |
| BMc297 | GO478016 | (AGA)4 | 102 | GCAGCGGAGGAGATGAGA | GATTGATCCATTCCCTTTCTG | 54 | NONE |
| BMc298 | GO478017 | (CAA)4 | 122 | CCTCCGTCCTCTCCTACGTC | GTAATCGGAGGAGGCGTTGT | 56 | NONE |
| BMc299 | GO478018 | (GAA)4(CAACT)3 | 146 | AGCGTGACAACACAGTAGGG | TGAGTTCAGGTGAGTAGAGCTGA | 54 | NONE |
| BMc300 | GO478019 | (AAG)5 | 82 | GCAAGTGATTCAGAAGTAAGAAG | CGGTTAACGGTCGTCACAG | 53 | gb|EU112623.1 |
| BMc301 | GO478020 | (TCG)4 | 97 | GAGAGTGGAGAATTAGGGTTTGA | CAGGATGCAGATCACCGTAA | 54 | gb|DQ503723.1 |
| BMc302 | GO478021 | (GAG)4 | 117 | TTTCGGGCGTGGCTTC | AGACGTCCGAGTTTCGTCAC | 56 | NONE |
| BMc303 | GO478022 | (ACC)5 | 118 | CACCACCACCGCCATATTA | GGACACGTTCTTCATTTCCAA | 55 | NONE |
| BMc304 | GO478023 | (TGC)5(AAGG)3 | 129 | TTGGAACTGGTTCTGAGTTGG | CCTTGTATTCATCTGACTGCTCA | 55 | gb|DQ503726.1 |
| BMc305 | GO478024 | (GT)6 | 100 | CCAGAGAGAAGGGAAGAGGAA | AACCCAAGTTGAGAAAGCTCC | 55 | NONE |
| BMc306 | GO478025 | (CCG)5 | 116 | GAAATCCTCCTCCACCACCT | GAACGGGTTTCTGCTTGATG | 55 | gb|DQ503718.1 |
| BMc307 | GO478026 | (CGA)6 | 104 | CGCACGATTACTGAAAGTTAGA | TCTGCCGAAACTAACACTCG | 53 | NONE |
| BMc308 | GO478027 | (CCT)4 | 104 | GTGGGCTGATTCTCTCCTCA | ACCAGTAGGTCTTCGCTTCG | 55 | NONE |
| BMc309 | GO478028 | (GAA)4 | 106 | AAGGCCCAGAGGGAGAAA | ACGACACACCAACGATCTGAAG | 56 | NONE |
| BMc310 | GO478029 | (CACAA)3 | 126 | AGTTTCGGAGGGAACACACA | TGGTGTTCTCTGGGTTTGGT | 55 | gb|AC148405.5 |
| BMc311 | GO478030 | (CGA)4 | 104 | AATCGCCTTGTTGTCCATGT | CTACAGCGGCATCTTCTTCC | 55 | emb|AJ867393.1 |
| BMc312 | GO478031 | (CCT)4(GAG)4 | 201 | GATCCGACGTCGTTTTGAG | GTGTTGATCCGTCATCAATGT | 54 | NONE |
| BMc313 | GO478032 | (TG)7 | 105 | CACCAAACCAATCACTCTACTGT | GGCGTCCAGAATGGAACT | 53 | gb|DQ503720.1 |
| BMc314 | GO478033 | (AG)11 | 165 | ACGCGTTCGGCCAGAGA | CCCGAATTCTGCACTACCAA | 58 | gb|DQ503775.1 |
| BMc315 | GO478034 | (GGA)4 | 112 | ACTCCAACAAATGCAGCAAA | TCGGAGCCTCCTGTCG | 54 | gb|DQ857254.1 |
| BMc316 | GO478035 | (CT)13 | 134 | CCCTTCCTTCTTCCCTTCAC | TTGCAGAGGAGTTGGAAACC | 55 | NONE |
| BMc317 | GO478036 | (CT)11 | 100 | CCCAACCAACCGTTTAAAGT | CAAATGAATGCAATGGAGGT | 54 | NONE |
| BMc318 | GO478037 | (TC)15 | 84 | CAAAAATTCTCTCTCTCTCTCTCTC | GGAGTCCATCAAACCCTAATATC | 52 | gb|DQ503777.1 |
| BMc319 | GO478038 | (CA)5(GCT)6 | 120 | CCCAGCCGGAAATAGAAACT | TCTGCACTGATTTGGTTGGT | 55 | dbj|AK285415.1 |
| BMc320 | GO478039 | (CA)5(GCT)6 | 80 | CCGCTCGAGAACACTTCTGT | TTCACCTCCTCACTTTGAACC | 55 | NONE |
| BMc321 | GO478040 | (CT)13 | 96 | CTTTCACTTCACTTGTGGTTCC | TGTTCCACTATCTCCCTGTTCA | 54 | gb|EU112619.1 |
| BMc322 | GO478041 | (CT)13 | 99 | GGCAACGTCCATAGGTTTCA | CAAAGACACAACAAGCGTGA | 55 | NONE |
| BMc323 | GO478042 | (TGC)4 | 80 | AATGACAAGGAGGGCAACAA | ATCCCAGCAACAGAGCAAAC | 55 | NONE |
| BMc324 | GO478043 | (GA)7 | 110 | CAGGATTCAGAGAGAAAAGGAG | GCAGCGACATGGTAGAAATG | 53 | NONE |
| BMc325 | GO478044 | (GTG)4 | 101 | AGGAGGCGGTTATGGAGGT | CTCGTCATCCCTCAGGAC | 54 | gb|EU112607.1 |
| BMc326 | GO478045 | (TCT)9(CCT)5 | 163 | CCATTGCTACCTTCACCAAA | GAGGGTTGGAATGTGGAGAG | 54 | dbj|AK285502.1 |
| BMc327 | GO478046 | (AC)5 | 117 | CCAAAACACAAACACACACA | TGGTTATCGGTTATCGGTTATG | 53 | gb|L38855.1| |
| BMc328 | GO478047 | (TC)5 | 142 | CTTCACCCGTGTTTCATTCG | GTTCCACCAGGCAGTCGT | 56 | NONE |
| BMc329 | GO478048 | (GGA)5(GGA)4 | 115 | TGATCTTCCACCAACTCATCA | GTAATCATAGTCACAGTAGGAAGGA | 52 | dbj|AK286018.1 |
| BMc330 | GO478049 | (TAA)8 | 102 | TCGATTGGGATTCTCTCTGAA | GCATAGCCCTACCACCTAGC | 54 | dbj|AK285457.1 |
| BMc331 | GO478050 | (CGG)5 | 87 | TTCAAGGAGGACGTTTGGTC | CCCAGCCTTCTCCCAAA | 55 | gb|EU112611.1 |
| BMc332 | GO478051 | (CAA)5 | 110 | TTCCCAACACAAACACCAAA | GAGGTGGAGGAAGAACTGGA | 55 | gb|AF372498.1| |
| BMc333 | GO478052 | (GTC)6 | 80 | CCGCTCGAGAACACTTCTGT | TTCACCTCCTCACTGTGAACC | 55 | dbj|AK285415.1 |
| BMc334 | GO478053 | (TCC)4 | 84 | CCTCTCGCTCATCTCGTT | AGCTGCGGGAGGGAGA | 54 | NONE |
| BMc335 | GO478054 | (CAA)5(TC)11 | 104 | CCAACAACAACAACAACATCC | CACATCAACAGCGCATACCT | 54 | NONE |
| BMc336 | GO478055 | (TC)5 | 94 | CGGAACTATCCCTGTTTCGT | GGCAAAGACCCAAATAGAAAGA | 54 | NONE |
| BMc337 | GO478056 | (CCG)4 | 115 | AATTGGGCTTCCACCACAT | CTATAATGGGCCAGGGCTTT | 55 | NONE |
| BMc338 | GO478057 | (CT)11 | 108 | TGGTGGAACAAACTCCTGTT | GACAGAACAACAGATAGACTGAAAT | 52 | NONE |
| BMc339 | GO478058 | (CT)11 | 128 | TCTAGGTTCACCAATTCACTCTTC | AGGGCAGTGAAATGAAACTT | 53 | NONE |
| BMc340 | GO478059 | (AC)5 | 135 | CACAAACTCAACACCCTCTCA | ACGTGCGAGACATCTCAACAG | 55 | NONE |
| BMc341 | GO478060 | (GGC)4(GTG)4 | 82 | TTATGGAGGTGGTGGTGGAT | CTCAGGAAGACCACCGTAGC | 55 | dbj|AK244324.1 |
| BMc342 | GO478061 | (AG)10 | 114 | CACACTCTAAAAAAGTAAGAGAGAG | CACCTGATGGCATGATTGAG | 52 | NONE |
| BMc343 | GO478062 | (GCA)5 | 150 | CCGCCGTTACTTCCAAACTA | GCCTGATTCTTCTGTACAGGTTT | 54 | NONE |
| BMc344 | GO478063 | (AG)7 | 81 | CAAAGGCATTGGGAAAATA | AAAAGGGTCAGAAGCGTCAA | 53 | NONE |
| BMc345 | GO478064 | (TC)6(AC)9 | 110 | CTCTTTTTCTCTTCATTTCTCTCTC | CCATCATAGAGGGCAACCAC | 53 | gb|EU112609.1 |
| BMc346 | GO478065 | (ACC4)(CCA)8 | 164 | CCTCTCTTTCTTTTCTGCTCTGA | GGGTGGTGATGGAGATGG | 55 | NONE |
| BMc347 | GO478066 | (TA)10(CT)9 | 136 | CATCACATGAATCTGTCACCAA | CAGGGTGCATAATTTAAGTCCA | 54 | dbj|AP007302.1 |
| BMc348 | GO478067 | (GAG)4 | 150 | GCATCACCACCCATTACTCTT | GTATGCTAGGCCTCCTGTGC | 54 | gb|EU112608.1 |
| BMc349 | GO478068 | (GTC)5 | 83 | TTGTGCTCCTCTGACAGTGC | GCCAGTAGCTTCGGTTCTCC | 56 | NONE |
| BMc350 | GO478069 | (CT)10(GAA)7 | 112 | TCTTCACACAACAAGTAGAACTTCG | CAGCTGGAAGGGAAGATGG | 55 | NONE |
| BMc351 | GO478070 | (CT)9 | 106 | AAACTTGTTCCGTCTGTTCC | GGGCTACAACCTCGGAGA | 53 | NONE |
| BMc352 | GO478071 | (TC)8 | 118 | TCTCTCTCTTACTCTTTCTCTCTCTC | AGAATTGAGAATTGTGCTGAGAA | 52 | NONE |
| BMc353 | GO478072 | (CT)5 | 94 | ACCTCTCACTTCTTCTAATCCTC | ATGATTAACAGAAAGGGATTCA | 50 | NONE |
| BMc354 | GO478073 | (ACA)4 | 129 | TCCCACCAATCTATCCAAGC | GCCTTCAGATCGGAGGATGT | 56 | gb|EU112613.1 |
| BMc355 | GO478074 | (GGGT)3 | 138 | GGGTCCTCTTTCTGTACATCG | CCCGAAAGAGCAACCAAAAG | 55 | NONE |
| BMc356 | GO478075 | (CAA)5 | 87 | TGCAGGGTTCACAGTCAGAG | AGAGAAAGCACTGCCCTACG | 55 | gb|EU112618.1 |
| BMc357 | GO478076 | (CAA)4 | 99 | CCACCATCAGCAACAACATC | AACTGGTTCCTTCTCAACCA | 54 | gb|AC123899.15 |
| BMc358 | GO478077 | (CAG)4 | 93 | CCCAGGTAAGCAACGATCAA | GCATTTGTTGTGGCTCCAAT | 56 | gb|DQ503755.1 |
| BMc359 | GO478078 | (CAA)5 | 118 | CAATGAGAACACTGGGACCTG | GACATGTGGAGGAATTGCTGT | 55 | NONE |
| BMc360 | GO478079 | (ACA)4 | 93 | CAGGCTACTGTTGCCCAGAT | CATTGCCTTCAAGAGTTGTT | 53 | NONE |
| BMc361 | GO478080 | (TCG)5(TC)5 | 144 | GCAATCGCAACAACAAATCT | GCAATCGCAACAACAAATCT | 54 | NONE |
| BMc362 | GO478081 | (CAG)7 | 116 | TGCAACAGCAGCAGCAAC | TTGTGGCAGCTGAGAAAGTT | 55 | NONE |
| BMc363 | GO478082 | (CCT)4 | 126 | AACGCCTACCTCTGCTCCTC | GTCGGCCTTGCATAGGAAC | 56 | gb|DQ371243.1 |
| BMc364 | GO478083 | (GAC)4 | 92 | ACCACTCCCACGTCGAAA | GTTGAGCAAGAGCCAGGAAG | 55 | NONE |
| BMc365 | GO478084 | (TC)8 | 126 | ACGCGTCCGGTTCTTTT | TCGATGAAACTGAGGGAACA | 54 | emb|AJ867393.1 |
| BMc366 | GO478085 | (TCT)4 | 82 | TTTGCCCAAACTCTCCATTC | GTTGGACACGAGGAGAGTGAA | 55 | gb|AC124951.19 |
| BMc367 | GO478086 | (GA)21 | 150 | GCTGGTGTTGTTCACCAAACT | CCTGTGAGCTATATCCTCGAAA | 54 | NONE |
| BMc368 | GO478087 | (TC)8 | 103 | TCCTCTCTCTCTCATTTCCAATC | AGTGCGTTTTGGTGAGAAGG | 55 | gb|DQ503754.1 |
